# Light Intensity Modulates the Functional Composition of Leaf Metabolite Groups and Phyllosphere Prokaryotic Community in Garden Lettuce (*Lactuca sativa* L.) Plants at the Vegetative Stage

**DOI:** 10.3390/ijms25031451

**Published:** 2024-01-25

**Authors:** Dedong Kong, Ziran Ye, Mengdi Dai, Bin Ma, Xiangfeng Tan

**Affiliations:** 1 Institute of Digital Agriculture, Zhejiang Academy of Agricultural Sciences, Hangzhou 310021, China; kongdd@zaas.ac.cn (D.K.); yezr@zaas.ac.cn (Z.Y.); daimd@zaas.ac.cn (M.D.); 2 Institute of Soil and Water Resources and Environmental Science, College of Environmental and Resource Sciences, Zhejiang University, Hangzhou 310058, China; bma@zju.edu.cn

**Keywords:** light intensity, leaf metabolites, garden lettuce, metagenomics, phyllosphere microbiome

## Abstract

Light intensity primarily drives plant growth and morphogenesis, whereas the ecological impact of light intensity on the phyllosphere (leaf surface and endosphere) microbiome is poorly understood. In this study, garden lettuce (*Lactuca sativa* L.) plants were grown under low, medium, and high light intensities. High light intensity remarkably induced the leaf contents of soluble proteins and chlorophylls, whereas it reduced the contents of leaf nitrate. In comparison, medium light intensity exhibited the highest contents of soluble sugar, cellulose, and free amino acids. Meanwhile, light intensity resulted in significant changes in the composition of functional genes but not in the taxonomic compositions of the prokaryotic community (bacteria and archaea) in the phyllosphere. Notably, garden lettuce plants under high light intensity treatment harbored more sulfur-cycling *mdh* and carbon-cycling *glyA* genes than under low light intensity, both of which were among the 20 most abundant prokaryotic genes in the leaf phyllosphere. Furthermore, the correlations between prokaryotic functional genes and lettuce leaf metabolite groups were examined to disclose their interactions under varying light intensities. The relative abundance of the *mdh* gene was positively correlated with leaf total chlorophyll content but negatively correlated with leaf nitrate content. In comparison, the relative abundance of the *glyA* gene was positively correlated with leaf total chlorophyll and carotenoids. Overall, this study revealed that the functional composition of the phyllosphere prokaryotic community and leaf metabolite groups were tightly linked in response to changing light intensities. These findings provided novel insights into the interactions between plants and prokaryotic microbes in indoor farming systems, which will help optimize environmental management in indoor farms and harness beneficial plant–microbe relationships for crop production.

## 1. Introduction

In indoor farms, heavy efforts have been made to optimize the environmental conditions for crops [1,2], but rarely for their symbiotic microbial friends. Instead, prokaryotic and fungal microbes are usually seen as potential threats to artificial agroecosystems [3]. It is common to utilize physical or chemical methods to eliminate microbial contamination in indoor farms [4,5], which, however, may cut off beneficial plant–microbe relationships. Immense studies have revealed the significance of the prokaryotic community in plant health and fitness [6,7]. However, the roles and adaptations of the prokaryotic microbes in indoor farming are far from being deciphered, which restricts the development of sustainable indoor agriculture. Therefore, it is necessary to look into the composition and function of the prokaryotic community as well as its interactions with crops.

Light condition is highly customized in indoor farming. Numerous studies have shown the impacts of light intensity and duration on the growth, biomass, pigment content, photosynthetic rate, and stress response of garden lettuce (*Lactuca sativa* L.) leaves [8,9]. Light quality (such as blue, green, and red) also has different impacts on the metabolic and transcriptional reprogramming of garden lettuce leaves [10,11]. The optimal light quality and intensity for garden lettuce growth depend on the cultivar and the environmental conditions [10]. The impacts of light intensity on plant–microbe interactions have only been uncovered recently. Light has both direct and indirect impacts on the phyllosphere microbiome. Photoreceptors in plants and microorganisms respond to narrow-bandwidth wavelengths of light, triggering specific internal responses that ultimately affect the composition and dynamics of the phyllosphere [12]. Recent research has shown that different wavelengths of light can significantly affect plant growth, prokaryotic and fungal diversity, and the overall functioning of the phyllosphere [13]. Light-induced plant responses include changes in hormonal levels, the production of secondary metabolites, and the release of volatile compounds, which influence interactions with microbes present in this environment. Microbes contribute by making essential elements biologically available for plants (nitrogen, phosphorus, and iron) as well as producing growth regulators promoting plant fitness, leading to a complex interplay between these two factors influencing each other’s behavior within this ecosystem. A recent study also showed that low photosynthetically active radiation perceived by *Arabidopsis thaliana* leaves modulates both leaf and root bacterial communities, indicating the interconnected effects of light on aboveground and belowground microbiota [14].

The composition and function of the prokaryotic community in indoor farms have only recently been studied through multi-omics methods. The phyllosphere, or aerial parts of plants, harbors diverse microbes in both epiphytic (the surface of a plant) and endophytic environments [15]. The phyllosphere is a unique habitat for microorganisms and is home to a diverse community of bacteria, archaea, viruses, fungi, microalgae, and other microorganisms [16,17,18]. It has been shown that Proteobacteria, Actinobacteria, and Bacteroidota are the dominant prokaryotic taxa in field-grown garden lettuce [19]. Another study found abundant Cyanobacteria in the phyllosphere of garden lettuce [20], possibly because of the high moisture during garden lettuce growth [21]. Phyllosphere prokaryotic communities undergo ubiquitous influence from both host plants and environmental variables [22,23]. Light intensity, for instance, has been shown to profoundly modulate the phyllosphere prokaryotic community in garden lettuce [24] and other plant species [13,25]. Considering that light intensity and spectra are ubiquitously modified to obtain optimal plant growth in indoor farms, it is critical to understand the mechanisms of interactions between plants and prokaryotic communities in the phyllosphere of garden lettuce. 

Apart from environmental conditions, leaf metabolic and physiological activities exert universal impacts on the phyllosphere prokaryotic community. Leaf primary and secondary metabolites can affect the plant prokaryotic community through a variety of mechanisms, including antimicrobial activity, stimulation of beneficial microbial growth, increased microbial diversity in the phyllosphere, reduction of pathogen populations, and induction of systemic resistance against pathogens [15,26]. The accessibility of simple carbon substrates, such as methanol, is among the most important factors affecting the colonization and fitness of leaf microorganisms [27]. During leaf colonization, bacterial species, including *Methylobacterium* spp. [28], and methanogenic archaea can consume methanol to improve their fitness [29]. In the phyllosphere of garden lettuce, the levels of leaf carbohydrates, calcium, and phenolic compounds significantly interact with the structure of the bacterial community [30], suggesting a plant–microbe interaction driven by substrate availability [27].

In this study, the influence of light intensity on the taxonomic and functional composition of the garden lettuce phyllosphere (defined as leaf surface and endosphere) prokaryotic community was deciphered through metagenomic methods. The leaf metabolite groups related to garden lettuce quality were also determined. We hypothesized that light intensity can modulate the phyllosphere prokaryotic community through the alteration of leaf metabolite groups.

## 2. Results

### 2.1. Light Intensity Modulated Leaf Metabolite Groups

**Figure 1:**
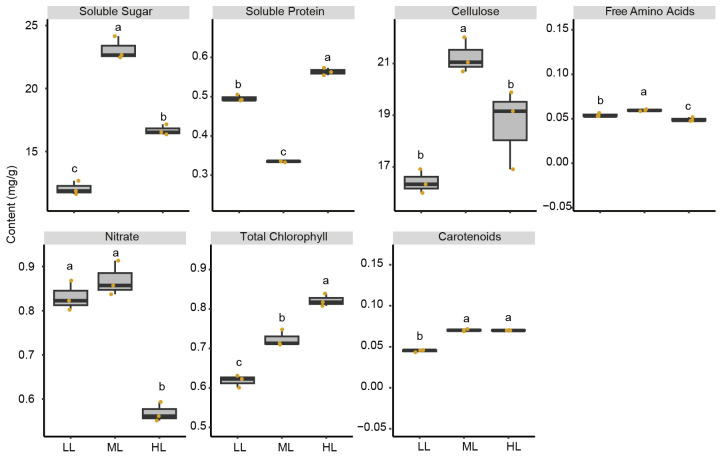
Leaf metabolite groups of garden lettuce (*Lactuca sativa* L.) under three light intensities. Contents were determined under low light (LL), medium light (ML), and high light (HL). Three biological replicates were determined for all metabolite groups. Box plots show median and quartiles, and points represent individual values of replicates (*n* = 3). Values were compared by one-way ANOVA and Tukey’s test (depicted as lower-case letters above the boxes). The contents were calculated as mg/g fresh weight.

Light intensity resulted in ubiquitous changes to all the measured leaf metabolite groups. Compared to the other two treatments, plants grown under high light intensity (HL) treatment showed significantly higher contents of soluble proteins (*p* = 5.26 × 10^−8^) and total chlorophylls (*p* = 2.62 × 10^−5^), but significantly lower leaf nitrate (*p* = 5.52 × 10^−5^) (one-way ANOVA and Tukey’s test) (Figure 1). In comparison, plants grown under medium light intensity (ML) treatment exhibited the highest contents of soluble sugar (*p* = 2.86 × 10^−6^), cellulose (*p* = 0.003), and free amino acids (*p* = 0.002) (Figure 1). In addition, the low light intensity (LL) treatment significantly reduced the leaf carotenoids (*p* = 2.4 × 10^−7^) (Figure 1).

### 2.2. Taxonomic Composition of the Phyllosphere Prokaryotic Community

**Figure 2:**
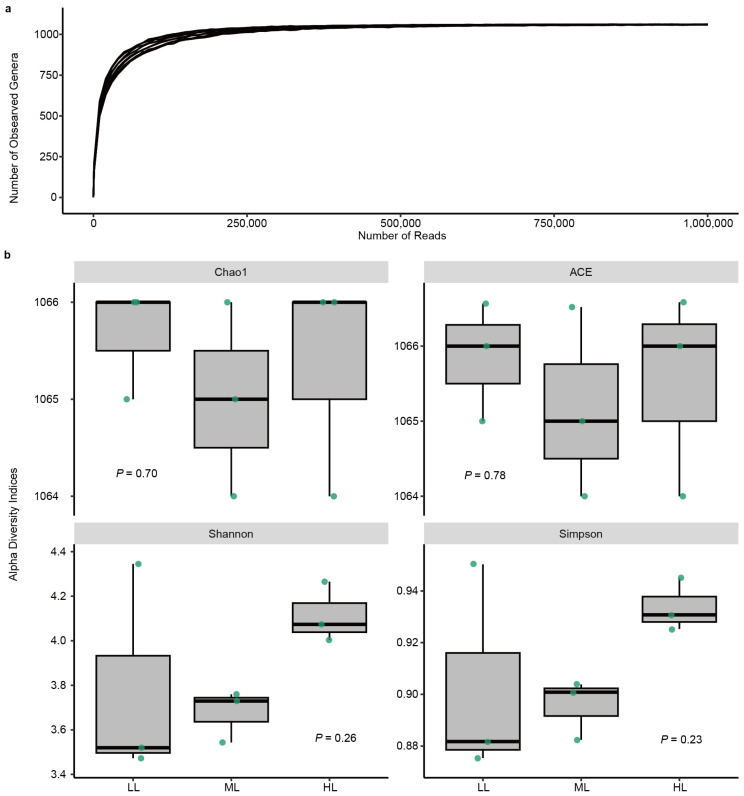
Prokaryotic diversity in the phyllosphere of garden lettuce (*Lactuca sativa* L.). (**a**) Rarefaction curve of samples at the genus level. Each line in the figure represents a sample plotting the number of observed prokaryotic genera against the number of reads. (**b**) Alpha diversity indices at the genus level. The *p*-values for one-way ANOVA are shown compared between low light (LL), medium light (ML), and high light (HL) treatments. Box plots show median and quartiles, and points represent individual values of replicates (*n* = 3).

Through metagenomic sequencing, the sequencing reads were assigned to 56 phyla, 111 classes, 249 orders, 449 families, 1066 genera, and 1891 species. The rarefaction curve indicated a sufficient coverage of species in the prokaryotic community (Figure 2a). Moreover, prokaryotic diversity (Shannon, Simpson, Chao1, and ACE indices) showed no difference compared between light treatments (one-way ANOVA, *p* > 0.05) (Figure 2b).

**Figure 3:**
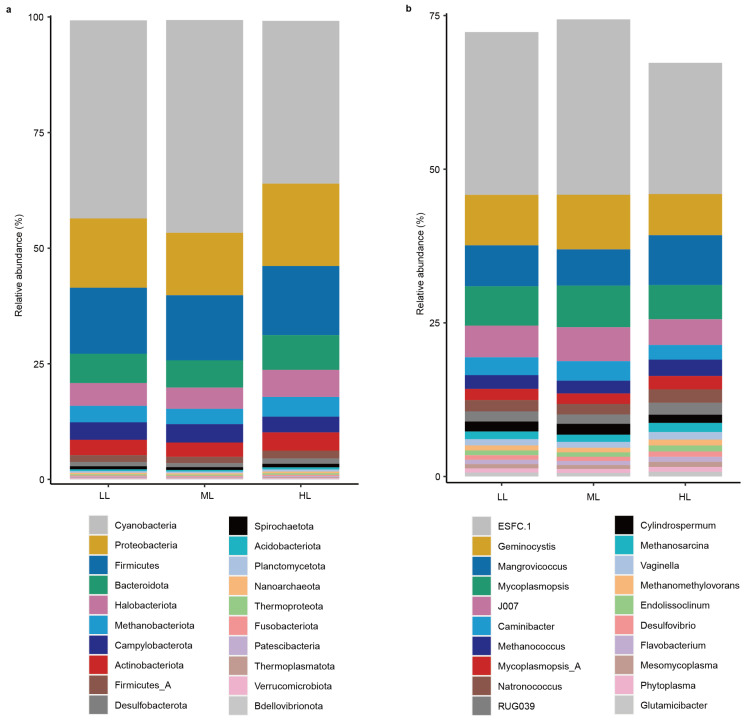
The prokaryotic composition in the garden lettuce (*Lactuca sativa* L.) phyllosphere. To increase clarity, only the 20 most abundant taxa are shown at the phylum (**a**) and genus (**b**) taxonomic levels, respectively. The average relative abundance of the replicates (*n* = 3) of three light intensities is displayed. LL: low light; ML: medium light; HL: high light.

The most abundant 20 phyla and 20 genera were visualized by stacked bar charts, respectively, to display their dominance in the phyllosphere prokaryotic community (Figure 3). The most abundant 20 prokaryotic phyla accounted for 99.3% relative abundance of all identified phyla (Figure 3a). Cyanobacteria (40.7%) dominated the phylum composition of the prokaryotic community, followed by the phyla Proteobacteria (15.9%), Firmicutes (14.5%), Bacteroidota (6.7%), and archaeal Halobacteriota (5.2%) (Figure 3a). At the genus level, the most abundant 20 prokaryotic genera accounted for 71.1% relative abundance of all identified genera (Figure 3b). The Cyanobacteria genera *ESFC.1* (25.1%), *Geminocystis* (7.7%), and *J007* (4.8%) were among the most abundant genera (Figure 3b), which were consistent with the phylum composition. The Bacteroidota genus *Mangrovimonas* (7.3%) and Firmicutes genus *Mycoplasmopsis* (6.2%) were also abundant in the phyllosphere prokaryotic community. In addition, *Methanococcus* (2.3%), *Natronococcus* (1.9%), *Methanosarcina* (1.3%), and *Methanomethylovorans* (0.9%) were the most abundant archaeal genera. Moreover, no effect of light intensity was detected compared between HL, ML, and LL treatments for the most abundant 20 phyla and genera (one-way ANOVA, *p* > 0.05).

### 2.3. Light Intensity Modulated the Functional Composition of the Phyllosphere Microbiome

**Figure 4:**
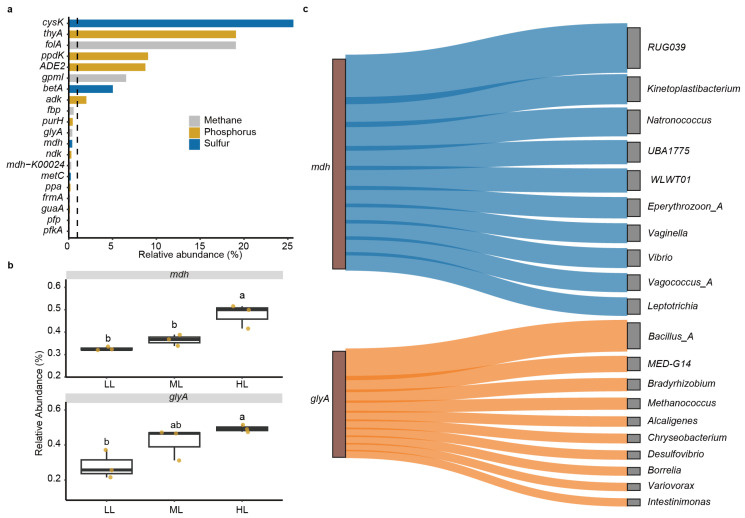
Prokaryotic functional genes in the garden lettuce (*Lactuca sativa* L.) phyllosphere. (**a**) The most abundant 20 prokaryotic genes. The genes were annotated against the databases containing prokaryotic genes involved in biogeochemical cycles of nitrogen, sulfur, phosphorus, and methane. (**b**) Comparison of gene relative abundance between light intensity treatments. Letters indicate Tukey’s post hoc comparisons after one-way ANOVA. Box plots show median and quartiles and points represent individual values of replicates (*n* = 3). Values were compared by one-way ANOVA and Tukey’s tests (depicted as lower-case letters above the boxes). LL: low light; ML: medium light; HL: high light. (**c**) Sankey diagram showing the sources of *mdh* and *glyA* genes from the prokaryotic genera. The assembled contigs harboring either of the two genes were counted, and their taxonomic assignment was analyzed. The width of edges is proportional to the read counts of metagenomic sequencing. To increase clarity, only the 10 most important prokaryotic genera are shown.

A total of 113 prokaryotic genes were annotated for the nitrogen, sulfur, phosphorus, and methane-cycling genes. The sulfur-cycling *cysK* (25.6%) and *betA* (5.0%); phosphorus-cycling *thyA* (19.1%), *ppdK* (9.0%), *ADE2* (8.7%), and *adk* (2.0%); and methane-cycling *folA* (19.1%) and *gpmI* (6.6%) genes showed high relative abundances over 1% among all prokaryotic genes (Figure 4a, Table 1). 

Among the 20 most abundant genes, one-way ANOVA and Tukey’s test identified two genes of *mdh* (*p* = 0.004) and *glyA* (*p* = 0.029) that were modulated by light-intensity treatments (Figure 4b). Garden lettuce plants under HL treatment harbored the highest relative abundance of *mdh* and *glyA* genes compared to the three treatments. The taxonomic sources of the *mdh* and *glyA* genes were analyzed by counting the assembled contigs harboring either of the two genes, respectively. The Actinobacteriota uncultured taxa *RUG039* (annotated by the Genome Taxonomy Database (GTDB) at the genus level) contributed the most proportion of *mdh* gene, followed by the genus *Kinetoplastibacterium* and the archaeal genus *Natronococcus* (Figure 4c). Moreover, the Firmicutes genus *Bacillus_A* harbors the most counts of the *glyA* gene, followed by the bacterial *MED-G14* (uncultured taxa at the genus level), the bacterial genus *Bradyrhizobium*, and the archaeal genus *Methanococcus*.

**Figure 5:**
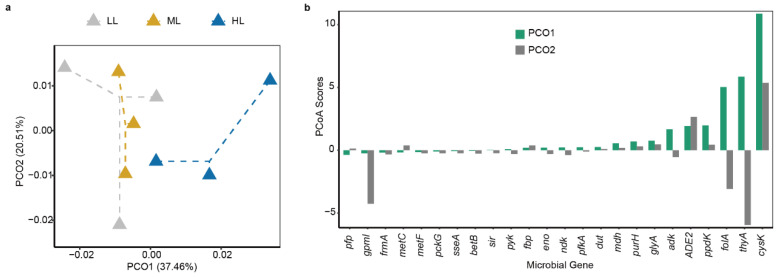
Dissimilarity of prokaryotic functional genes in the garden lettuce (*Lactuca sativa* L.) phyllosphere. (**a**) Principal coordinate analysis of prokaryotic genes. The first two principal coordinates (PCOs) were extracted and visualized. (**b**) Contributions of prokaryotic genes to the top two PCOs. ‘Species scores’ of each of the prokaryotic genes were calculated for the first two PCOs, respectively. To increase clarity, only the genes showing ‘species scores’ greater than 0.2 or less than −0.2 for either of the two PCOs were included. LL: low light; ML: medium light; HL: high light.

The first two ordination axes of principal coordinate analysis (PCoA) explained 57.97% of the variation of dissimilarity based on the relative abundance of functional genes (Figure 5). The gene dissimilarity of LL and ML treatments overlapped but was distinct from that of HL treatment (Figure 5a). To disclose the importance of each of the genes to overall gene dissimilarity, we analyzed the ‘species scores’ of PCoA ordination. The *cysK*, *thyA*, and *folA* genes contributed most to the first ordination axis, and the *thyA*, *cysK*, and *gpmI* genes contributed most to the second ordination axis (Figure 5b). Noticeably, these genes were also among the most abundant prokaryotic genes (Figure 4a). 

### 2.4. Correlations between Leaf Metabolite Groups and Prokaryotic Functional Genes

**Figure 6:**
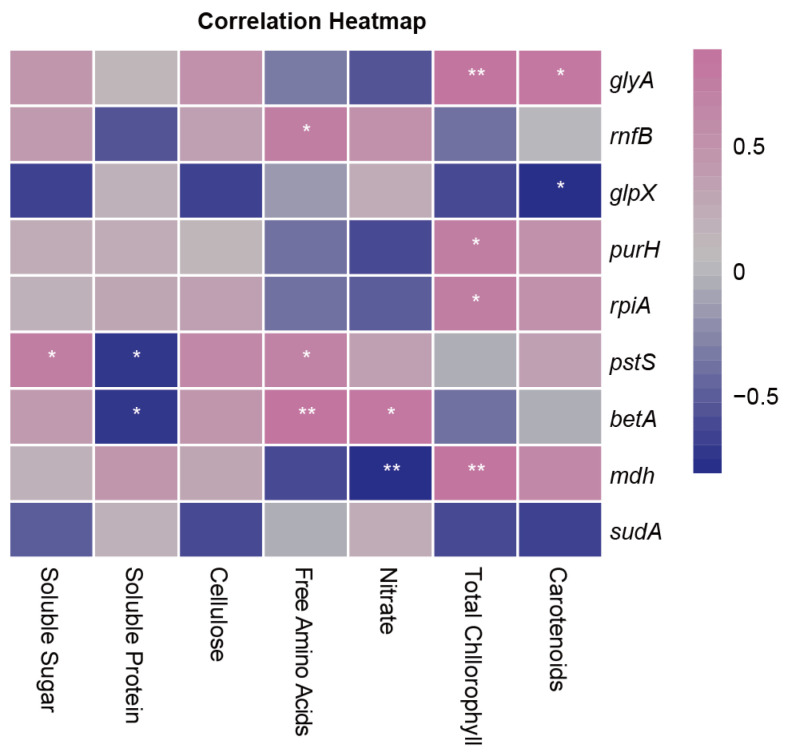
Correlations between leaf metabolite groups and prokaryotic functional genes in the garden lettuce (*Lactuca sativa* L.) phyllosphere. Only significant correlations are shown in the blocks. Positive correlations are indicated as pink and negative ones as purple. The asterisks in the blocks indicate the significance levels: * *p* ≤ 0.05; ** *p* ≤ 0.01.

Correlation tests were performed to discover the functional connections between leaf metabolite groups and prokaryotic genes. Nine prokaryotic genes were significantly correlated with at least one leaf metabolite group (Figure 6). Notably, leaf total chlorophyll contents showed positive correlations with *glyA*, *mdh*, and two phosphorus-cycling genes (*purH* and *rpiA*). The total free amino acid content was positively correlated with the phosphorus-cycling *pstS* and sulfur-cycling *betA* genes, but the soluble protein content was negatively correlated with these two genes. In addition, leaf carotenoid content was positively correlated with the *glyA* gene but negatively with the methane-cycling *glpX* gene.

**Figure 7:**
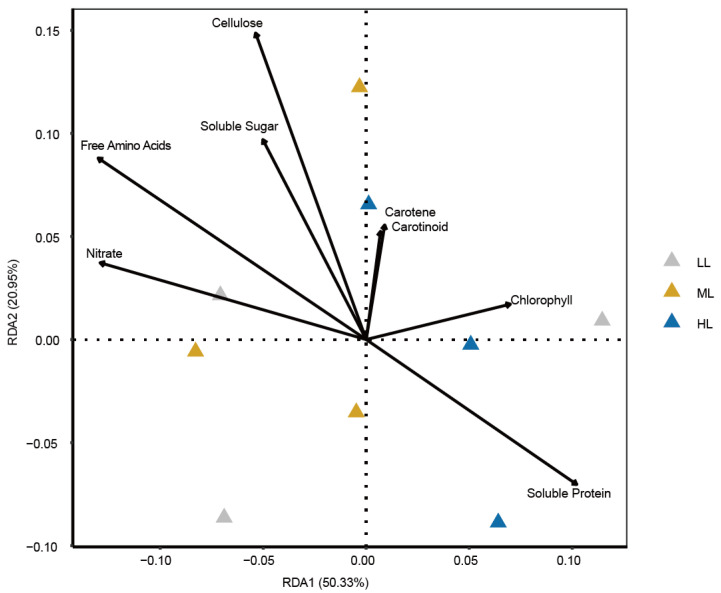
Effects of leaf metabolite groups on prokaryotic gene dissimilarity in the garden lettuce (*Lactuca sativa* L.) phyllosphere. Redundancy analysis (RDA) was performed based on the Bray–Curtis dissimilarity of prokaryotic functional genes. The length of the arrows indicated the effects of each metabolite group. The same direction of the arrows indicates positive correlations, and the opposite direction indicates negative correlations. LL: low light; ML: medium light; HL: high light.

The eight determined leaf metabolite groups significantly explained the dissimilarity of prokaryotic genes (redundancy analysis, *p* = 0.0002) (Figure 7). Leaf-soluble protein, total free amino acids, and cellulose contents were the most outstandingly determined factors interacting with the dissimilarity of prokaryotic genes. Moreover, the arrow of leaf soluble protein pointed in different directions compared to total free amino acids and cellulose, suggesting their negative correlations (Figure 7).

## 3. Discussion

### 3.1. Leaf Metabolite Groups under Varied Light Intensity

The impacts of light intensity on garden lettuce leaf growth and metabolism are straightforward since it directly alters photosynthesis [31,32]. Meanwhile, the mechanisms are complicated considering the ubiquitous interplays between photosynthetic products and other metabolic pathways. In this study, light intensity significantly influenced all the examined leaf metabolite groups in plants, which are often used as indicators for plant growth and the quality of leafy vegetables [33,34]. In this study, the increase in light intensity from ~170 μmol m^−2^ s^−1^ to ~240 μmol m^−2^ s^−1^ leads to the increase in the content of leaf total chlorophyll. Strong light may reduce leaf total chlorophyll content because of chlorophyll degradation [35] and the inhibition of chloroplast formation [31]. However, the leaf chlorophyll accumulation can be induced by the moderate increase in light intensity under low light before being inhibited by high light intensity [36]. Therefore, the increased total chlorophyll content under the high light treatment may be derived from the relatively narrow range of light treatments in this study. Our results showed that high light intensity induced carotenoid accumulation. High light intensity induces the accumulation of three carotenoids, violaxanthin, antheraxanthin, and zeaxanthin in a meta-analysis with 760 plant species [37], which may increase the flexibility of plant cells under sharply changing light conditions. However, other carotenoids, such as lutein and β-carotene, are less responsive to light intensity [38], and thus the determinations of different carotenoids are needed in follow-up studies to further disclose their response to light intensity. Moreover, high light intensity reduced leaf nitrate in the samples. Nitrate acts as the nitrogen source for proteins, chlorophyll, and DNA/RNA. Other studies also find that high light intensity leads to a decrease in leaf nitrate content in different plant species [37,39], possibly due to the stimulated conversion of nitrate to amino acids and other organic compounds under high light conditions [39]. Moreover, medium light intensity showed the highest contents of soluble sugar, cellulose, and free amino acids. A previous study also observed the reduced leaf soluble sugar and free amino acids in garden lettuce plants under high light intensity [34]. This indicated that a proper light intensity was needed for the stock of sugar and amino acids in garden lettuce plants [40].

### 3.2. Taxonomic and Functional Composition of the Phyllosphere Prokaryotic Community

The phyllosphere provides a unique and dynamic environment for microorganisms, characterized by factors such as nutrient limitation, exposure to sunlight, and interactions with plant metabolism [27]. Thus, the prokaryotic microbes in the phyllosphere were interactively influenced by environmental and plant factors. Cyanobacteria were the most abundant in the garden lettuce phyllosphere prokaryotic community. Other studies also found abundant Cyanobacteria in garden lettuce leaves [20,41] and the phyllosphere environments of other species with high moisture [21]. Cyanobacteria species conduct oxygenic photosynthesis and nitrogen fixation in the phyllosphere [42]. Cyanobacteria have been found to respond to light changes [43]. The high abundance of Cyanobacteria in the studied samples suggests its adaptation in the phyllosphere. Moreover, three genera of archaeal methanogens, *Methanococcus*, *Methanosarcina*, and *Methanomethylovorans*, were among the most abundant prokaryotic genera. These genera in the phyllosphere can produce methane gas by utilizing simple carbon sources, including methanol. The results were in agreement with other reports on the wide existence of archaeal methanogenesis in the phyllosphere [29,44]. The shaping factors for the composition of the phyllosphere microbial community in garden lettuce plants include the cultivation methods. It has been shown that the phyllosphere bacterial communities of garden lettuce plants differed between field-grown and laboratory-grown plants [45]. Moreover, soil and hydroponically grown plants may also harbor differential phyllosphere microbial communities [46] since growth substrates serve as important reservoirs of microbial transmission to the canopy [47]. Therefore, different planting methods can be included in the follow-up studies to further reveal the assembly mechanism of the phyllosphere microbial community in garden lettuce. 

Metagenomic analyses also disclosed the functional composition of the phyllosphere prokaryotic community. This study comprehensively deciphered the links of prokaryotic genes to the biogeochemical cycles of sulfur, phosphorus, methane, and nitrogen in the phyllosphere of garden lettuce plants, which was rarely studied in indoor-grown garden lettuce plants. As fundamental functional units, prokaryotic genes are ubiquitously involved in nutrient cycling, which is essential for both prokaryotic survival and plant growth [27]. In our results, the sulfur-cycling genes *cysK* (cysteine synthase) and *betA* (Oxygen-dependent choline dehydrogenase) were among the most abundant prokaryotic genes in the phyllosphere prokaryotic community. The *cysK* is involved in the “Link between inorganic and organic sulfur transformation” pathway, and *betA* is involved in the “organic sulfur transformation” pathway (Table 1), which are both key microbial processes mediating the microbial utilization of sulfur compounds [48]. In the nutrient-limited phyllosphere environment, the ability to use sulfur compounds, including organosulfonic compounds, was crucial for microbial survival [29].

The *folA* and *gpmI* genes were the most abundant carbon-cycling genes in the phyllosphere, both of which were involved in the serine cycle. The *folA* gene encodes a dihydrofolate reductase that plays an essential role in forming a key coenzyme in one-carbon transfer pathways (Table 1) [49]. Likely, the *gpmI* gene encodes a phosphoglycerate mutase and contributes to glycolysis and gluconeogenesis (Table 1) [50]. Thus, the abundant *folA* and *gpmI* genes likely reflect the importance of methylotrophy in the phyllosphere prokaryotic community.

### 3.3. Impacts of Light Intensity on Phyllosphere Prokaryotic Genes and Interactions with Leaf Metabolite Groups

We observed no influence of light intensity on prokaryotic taxonomic composition or diversity. Instead, light intensity greatly modulated the composition of prokaryotic functional genes. Among the most abundant functional genes, light intensity promoted the relative abundance of *mdh* and *glyA* genes, which are involved in the sulfur and methane cycles, respectively [48,51]. 

The *glyA* gene showed distinct relative abundances under different light intensities. Like the *folA* and *gpmI* genes mentioned above, the *glyA* gene is also involved in the serine cycle (Table 1), which plays a crucial role in methylotrophy [52]. Moreover, the relative abundance of the *glyA* gene was positively correlated with the contents of total chlorophyll and carotenoids. Considering that the contents of these pigments were increased in HL treatments compared to LL treatment, our results suggested a higher potential of methylotrophy under HL conditions than lower light intensities. Further analysis showed that the taxa of *Bacillus_A*, *Bradyrhizobium*, and the uncultured *MED-G14* at the genus level mainly contributed to the *glyA* abundance, of which the former two genera were repeatedly found to be plant-growth-promoting microbes [53,54]. Moreover, the *glyA* gene was essential in the biosynthesis of glycine from the one-carbon substrate in *Bradyrhizobium* species [55].

The *mdh* gene is involved in the ‘organic sulfur transformation’ pathway of the sulfur cycle. Unlike the most abundant sulfur-cycling genes of *cysK* and *betA*, our results showed that the relative abundance of *mdh* can respond to varied light intensities. Consistent with the *glyA* gene, the *mdh* gene also showed higher abundance at higher light intensities than the LL treatment and was positively correlated with leaf total chlorophyll content. Interestingly, *mdh* abundance was negatively correlated with leaf nitrate content, possibly because of the consumption of nitrate for photosynthetic metabolism under high light intensity [37]. The uncultured *RUG039* at the genus level is rarely reported in other studies. In our results, *RUG039* contributed to the highest proportion of *mdh* genes, suggesting its important role in phyllosphere sulfur metabolism.

Leaf soluble protein, total free amino acids, and cellulose contents were the most outstandingly determined factors interacting with the dissimilarity of prokaryotic genes, suggesting the importance of organic compound metabolism to phyllosphere prokaryotic microbes. Consistently, another study also found the shaping effects of leaf morphological and chemical traits on the phyllosphere bacterial community in garden lettuce plants [30]. However, our results showed that the determined leaf metabolite groups mainly drive the variation of functional genes instead of the taxonomic composition. These results indicate that the composition of prokaryotic genes can serve as functional traits in the response of the phyllosphere prokaryotic community to changing light intensity. In nutrient-limited phyllosphere environments, the utilization of leaf metabolites is crucial for microbial survival. However, on the other hand, these metabolite groups are also the major nutritional compounds provided by indoor-farming crops. Thus, the interactions between crops and the microbiome should be further studied to harness beneficial microbes and minimize detrimental impacts [56]. This study implied a possible technical pathway to modulate plant–microbe interactions through light regulation in garden lettuce production. However, because metagenomic sequencing is limited to the abundance of microbial genes but lacks functional information on expression, further studies on gene transcription and protein expression should be conducted to reveal a comprehensive impact of light intensities on microbial functioning in the garden lettuce phyllosphere.

## 4. Materials and Methods

### 4.1. Plant Materials and Sampling

**Figure 8:**
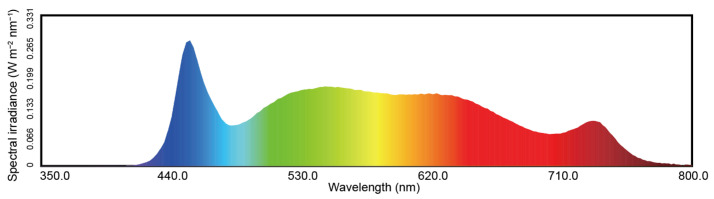
Spectra of the LED lights used in this study. Colors indicate different wavelengths. The spectral irradiance shown in the figure was set for the low light treatment and adjusted for other light intensities.

Romaine-type garden lettuce (*L. sativa* L. cv. ’Ideal-205’) (Ideal Agriculture Technology, Nanjing, China) plants were grown in a growth chamber supplied with 16/8 h (light/dark) cycle, 25/20 °C (day/night) temperature, and 85% relative humidity. Cold white LED light was provided with a correlated color temperature of about 5000 K (Figure 8). Preliminary experiments showed garden lettuce plants grew well under the light intensity of 24,000 lx, and in this experiment, low light (LL), medium light (ML), and high light (HL) intensities were set up as 20,000 lx (173.26 ± 3.51 μmol m^−2^ s^−1^), 24,000 lx (204.41 ± 4.97 μmol m^−2^ s^−1^), and 28,000 lx (242.57 ± 3.93 μmol m^−2^ s^−1^), respectively (PLA-30, Everfine Corporation, Hangzhou, China). The seeds were germinated and grown on moss peat soil (Pindstrup Mosebrug A/S, Ryomgaard, Denmark). No extra nutrients were supplied throughout the experiment. Each seedling was transplanted to a square pot (100 mm top diameter × 75 mm bottom diameter × 85 mm depth) after the second true leaf had fully unfolded. Sixteen plants were grown at each light intensity for 21 days. The plants were at the vegetative stage at the end of the experiments. After that, three replicates of healthy plants from each treatment were chosen randomly and taken as independent biological samples for metagenomic sequencing and leaf metabolite group determination, respectively. 

### 4.2. Metagenomic Sequencing and Prokaryotic Taxonomy

Leaf samples were stored at −80 °C before DNA extraction. The samples were untreated to keep the microorganisms from both the leaf surface and endosphere. Total DNA was extracted with a FastDNA Spin Kit (MP Bio, Santa Ana, CA, USA). Paired-end shotgun sequencing (2 × 150 bp) was performed at PersonalBio (Shanghai, China) using an Illumina NovaSeq PE150 platform (Illumina, San Diego, CA, USA). Raw sequences were quality-controlled with the Trimmomatic-v0.39 software (Bolger et al., Jülich, Germany) [57]. Plant genomic reads were identified and removed by aligning the trimmed reads to garden lettuce reference genome Lsat Salinas v11 (NCBI RefSeq assembly: GCF_002870075.4) using Bowtie2-2.3.2 (Langmead and Salzberg, College Park, MD, USA). Clean reads were assembled into contigs with the Megahit v1.2.9 software (Li et al., Hongkong, China). Bacterial and archaeal taxonomic annotations were performed with the CLARKSCV1.2.6.1 software (Ounit et al., Riverside, CA, USA) against the GTDB database R202 [58]. Clean reads were then mapped to contigs with the Bowtie2-2.3.2 software and sorted in Samtools v1.9 (Danecek et al., Hinxton, UK). After that, the abundance table was generated with the “idxstats” command in Samtools. 

The prokaryotic read counts were grouped at the taxonomic levels of domain, phylum, class, order, family, genus, and species, respectively. The relative abundance was calculated by dividing the number of reads aligned to prokaryotic taxa by the total number of reads of a sample. The average relative abundance of prokaryotic taxa in each of the three light treatments was used to generate stacked bar charts for the prokaryotic composition at phylum and genus levels, respectively. For clarity purposes, only the 20 most abundant phyla or genera were shown in the bar charts. Shannon, Simpson, Chao1, and ACE diversity indices were calculated at the genus level with the ‘diversity’ function in the vegan R package.

### 4.3. Functional Annotations of Prokaryotic Sequences

The prokaryotic sequences were searched using the BLAST algorithm of local alignment against four databases, NCycDB [59], SCycDB [48], MCycDB [51], and PCyCDB [60], to disclose the functional potentials of prokaryotic genes in the biogeochemical cycles of nitrogen, sulfur, methane, and phosphorus, respectively. In each of the databases, prokaryotic genes were annotated with their gene names and their biological pathways. For instance, the *cysK* gene is annotated as ‘cysteine synthase’ and is involved in the ‘link between inorganic and organic sulfur transformation’ pathway in the sulfur cycle. The Diamond v0.9.22 software (Buchfink et al., Tübingen, Germany) was used for sequence search with the “blastx” command. Thereafter, a PERL script (https://github.com/qichao1984/NCyc/blob/master/NCycProfiler.PL, accesed on 31 August 2021) was employed to calculate the abundance of prokaryotic genes. The counts of functional genes were normalized through a random subsampling method to eliminate the effects of differences in the number of sequenced reads across samples [61].

The abundance tables of genes annotated to the four databases stated above were merged into one, and gene relative abundance was calculated by dividing the number of reads aligned to a gene by the total number of annotated reads of a sample. The average relative abundance of the 20 most abundant genes in all samples was visualized in a bar chart and was further compared between three light treatments by one-way ANOVA and Tukey’s tests; in the comparisons with significant (*p* < 0.05) differences (*mdh* and *glyA* in this study), the relative abundance of genes in three light treatments was visualized by box plots. 

To disclose the taxonomic sources of the *mdh* and *glyA* genes, the assembled contigs harboring either of the two genes were counted, and their taxonomic assignments were visualized by Sankey diagrams with the ‘sankeyNetwork’ function in the networkD3 package for R.

The Bray–Curtis dissimilarity of prokaryotic genes was calculated with the ‘vegdist’ function based on the relative abundance table of the microbial genes. Gene dissimilarity was visualized by the PCoA ordination with the ‘cmdscale’ function in the vegan package. The ‘species scores’ were added to the PCoA by the ‘add.spec.scores’ function in the BiodiversityR package, which was aimed at evaluating the contribution of each prokaryotic gene to the principal coordinates (ordination axes).

### 4.4. Leaf Biochemical Analysis

Standard samples of sucrose, bovine serum albumin (BSA), cellulose, KNO_3_, and L-Leucine (Sangon Biotech, Shanghai, China) were diluted and determined following the methods below to build standard curves for the determinations of total soluble sugar, total soluble protein, cellulose, nitrate, and free amino acids, respectively. The contents were calculated as mg/g fresh weight.

The total soluble sugar content of leaf samples was determined by the anthrone method [34]. Fresh leaf sample (0.5 g) was boiled in 10 mL of distilled water for 30 min. The supernatant (0.1 mL) was transferred to the solution with 1.9 mL of distilled water, 0.5 mL of a 2% solution of anthrone in ethyl acetate (Sangon Biotech, Shanghai, China), and 5 mL of sulfuric acid. The total soluble sugar was detected by a UV-VIS spectrophotometer (T6 new century, Beijing General Analytic Instrument Ltd. Co., Beijing, China) at 630 nm wavelength.

The content of soluble protein in leaf samples was determined by the Coomassie brilliant blue colorimetric method [34]. A total of 1.0 g of leaf sample was ground in a mortar with 2 mL of distilled water. The homogenate was rinsed with 6 mL of distilled water and transferred to a centrifuge tube. The sample was left at room temperature (20–25 °C) for 0.5–1 h for full extraction and then centrifuged at 4000 r/min for 20 min. The supernatant (0.05 mL) was transferred to a volumetric flask and the volume was added to 10 mL with 5 mL of Coomassie brilliant blue G-250 solution (Sangon Biotech, Shanghai, China, 0.1 g L^−1^) and distilled water. The soluble protein was detected by a UV-VIS spectrophotometer at 595 nm.

The total free amino acid content of leaf samples was determined by the ninhydrin chromogenic method. A total of 0.5 g of leaf sample was ground with 5 mL of 10% acetic acid (Sinopharm Chemical Reagent Co. Ltd., Shanghai, China). A total of 2 mL of supernatant was diluted with 0.2 M Na-acetate buffer (pH 5.4) (Sangon Biotech, Shanghai, China) to 25 mL, and 2 mL of the solution was boiled for 15 min. During the determination, 2 mL of sample diluent was mixed with 0.1 mL of ascorbic acid (0.1%) and 3 mL of hydrated ninhydrin solution (Sangon Biotech, Shanghai, China), and the free amino acid content was determined by a UV-VIS spectrophotometer at 580 nm.

The content of leaf cellulose was determined by anthrone sulfate colorimetry [62]. Leaf samples (0.5 g) were hydrolyzed to glucose with 15–20 mL of 60% sulfuric acid (Sinopharm Chemical Reagent Co. Ltd., Shanghai, China). Then, the monosaccharide was dehydrated to furfural compounds with the action of sulfuric acid. The cellulose content was determined by a UV-VIS spectrophotometer at 620 nm wavelength with 0.5 mL of a 2% solution of anthrone in ethyl acetate and 5 mL of sulfuric acid.

The leaf nitrate was extracted with ammonia buffer (20 mL of 36–38% HCl and 50 mL of 25–28% ammonium hydroxide in 1 L of distilled water, pH 9.6–9.7) (Sinopharm Chemical Reagent Co. Ltd., Shanghai, China) and nitrate was determined by a UV-VIS spectrophotometer at 219 nm. 

The total chlorophyll and carotenoids in 0.2 g of leaf sample were extracted with 95% ethanol and determined by a UV-VIS spectrophotometer at 665 nm, 649 nm, and 470 nm [63]. 

### 4.5. Statistics

The statistical analyses were performed in R 4.2.1 (R Core Team, Vienna, Austria). Means were compared between the light treatments by one-way ANOVA and Tukey’s test in the agricolae R package. Correlation tests were performed with the ‘corr.test’ function in the psych package with the ‘Pearson’ method. The ‘pheatmap’ function in the pheatmap R package was employed to generate a correlation heatmap. Redundancy analysis was performed with the ‘rda’ function in the vegan package to evaluate the effects of leaf metabolite groups on prokaryotic gene dissimilarity.

## 5. Conclusions

Overall, the effects of light intensity on garden lettuce leaf metabolite groups and the phyllosphere prokaryotic community were examined in this study. The findings revealed that light intensity had a substantial impact on the content of leaf metabolite groups but not on the taxonomic composition and diversity of the phyllosphere prokaryotic community. Light intensity, on the other hand, altered the functional content of prokaryotic genes, particularly those involved in methane cycling. Furthermore, the functional linkages between plant metabolism and the phyllosphere prokaryotic community were shown by correlations between leaf metabolite groups and prokaryotic genes. These findings imply that light intensity has a significant impact on plant–microbe interactions in indoor farming systems. Considering that plant developmental status and cultivation methods (soil and hydroponics) can also affect the functional composition of the phyllosphere microbiome and its interaction with light intensity, more research is needed to understand the processes and consequences of light-induced plant–microbe interactions on the development and product quality of indoor-grown garden lettuce. 

## Figures and Tables

**Table 1:** Functional annotations of the 20 most abundant prokaryotic genes in the garden lettuce (*Lactuca sativa* L.) phyllosphere. Metagenomic reads were annotated to the biogeochemical cycling gene databases of sulfur (S), phosphorus (P), and methane (M).

Gene	Annotation	Pathway	Cycle
*metC*	Cystathionine beta-lyase	Link between inorganic and organic sulfur transformation	S
*cysK*	Cysteine synthase	Link between inorganic and organic sulfur transformation	
*mdh*	Malate dehydrogenase	Organic sulfur transformation	
*betA*	Oxygen-dependent choline dehydrogenase	Organic sulfur transformation	
*ppa*	Inorganic pyrophosphatase	Oxidative phosphorylation	P
*adk*	Adenylate kinase	Purine metabolism	
*guaA*	GMP synthase (glutamine-hydrolyzing)	Purine metabolism	
*ADE2*	Phosphoribosylaminoimidazole carboxylase	Purine metabolism	
*purH*	Phosphoribosylaminoimidazolecarboxamide formyltransferase/IMP cyclohydrolase	Purine metabolism	
*ndk*	Nucleoside-diphosphate kinase	Purine metabolism/Pyrimidine metabolism	
*thyA*	Thymidylate synthase	Pyrimidine metabolism	
*ppdK*	Pyruvate, orthophosphate dikinase	Pyruvate metabolism	
*frmA*	S-(hydroxymethyl)glutathione dehydrogenase/alcohol dehydrogenase	Oxidation of formaldehyde	M
*pfkA*	6-phosphofructokinase	RuMP cycle	
*fbp*	Fructose–1,6-bisphosphatase I	RuMP cycle	
*pfp*	Pyrophosphate–fructose 6-phosphate 1-phosphotransferase	RuMP cycle	
*folA*	Dihydrofolate reductase	Serine cycle	
*glyA*	Glycine hydroxymethyltransferase	Serine cycle	
*mdh-K00024*	Malate dehydrogenase	Serine cycle	
*gpmI*	Phosphoglycerate mutase	Serine cycle	

## Data Availability

The raw metagenomic sequencing data have been deposited to the Genome Sequence Archive (GSA) database under the accession number CRA012153.
